# Editorial: Shock and resuscitation

**DOI:** 10.3389/fphys.2023.1231691

**Published:** 2023-07-07

**Authors:** Lusha Xiang, John S. Clemmer, Belinda H. McCully

**Affiliations:** ^1^ United States Army Institute of Surgical Research, San Antonio, TX, United States; ^2^ Department of Physiology, University of Mississippi Medical Center, Jackson, MS, United States; ^3^ Department of Basic Medical Sciences, College of Osteopathic Medicine of the Pacific-Northwest, Western University of Health Sciences, Lebanon, OR, United States

**Keywords:** hemorrhage, volume, hemodynamics, coagulation, metabolism

Shock is a common but fatal complication and represents a major preventable cause of death in trauma patients. Controversy still exists concerning using appropriate types and timing of resuscitation fluids, allowing permissive hypotension, and preventing coagulopathy and multiple organ failure in severely injured patients. The current Research Topic of *Shock and Resuscitation* welcomed submissions from clinical and basic science research. This Research Topic includes summaries on different types of shock-associated injuries including their etiology, mechanisms, pathophysiology. Also included are novel potential therapeutics and techniques targeting patient survival, multiple organ failure, abnormal coagulation, tissue tolerance to ischemia, and optimal strategies for hemorrhage resuscitation.

In theory, the most effective antishock treatment is one that targets the root mechanism of shock—lack of tissue perfusion. Such treatments should improve tissue perfusion at microcirculatory levels rather than primarily focusing on systemic blood pressure (Naumann et al., 2016; Dubin et al., 2020). Notably, microcirculatory responses to shock or resuscitation are not always connected to systemic hemodynamics, and, likewise, macrohemodynamic variables cannot always be relied upon to monitor the outcome of shock conditions. With this understanding, blood flow in vital organs needs to be considered as a routinely measured index to evaluate severity of shock and efficacy of treatment (Elansary et al.; Yoshimoto et al.). For example, Patel et al. demonstrated that cerebral blood flow during cardiac massage in pigs undergoing cardiac arrest remained severely compromised, and the effect of cardiac massage on cardiac flow was absent until volume resuscitation was provided. These measurements suggest that open cardiac massage is a poor technique to achieve adequate vital organ perfusion, and volume expansion is still the priority when considering critical restoration of vital organ perfusion and energy supplies.

Indeed, recovery of organ function from hemorrhagic shock tightly correlates with restoration of ATP levels in tissues (Crowell and Smith, 1964; Clowes and Hershey, 1971). Notably, adding metabolic substrates alone to prevent cellular fatigue during prolonged shock without improving oxygen supply is likely an effort in vain. For example, without oxygen, lipid oxidation is suppressed, and glucose would rapidly be consumed via anaerobic glycolysis to meet ATP demands. If ischemia is prolonged, accumulation of intermediates of the tricarboxylic acid cycle (TCA) further shifts pyruvate metabolism towards other anaerobic catabolism such as lactate and hexose-monophosphate pathways, resulting in exacerbated acidosis and impaired redox balance. There is evidence that glucose injections following prolonged shock can exacerbate the accumulation of lactate without altering any TCA metabolites (D’Alessandro et al., 2015). Pyruvate-based fluids have been proposed as resuscitative fluids because of their anti-inflammatory and antioxidant properties that protect against organ dysfunction and metabolic disturbances in numerous preclinical studies with various pathogenic injuries (Zhou). However, it should be realized that the beneficial effects of pyruvate exclusively depend on sufficient tissue perfusion.

Hypotensive resuscitation with antishock compounds for volume expansion was studied for golden hour extension in the case where full resuscitation is unavailable during prehospital care (Parrish et al., 2016; Xiang et al., 2023). A low dose of Centhaquine was shown to cause venous constriction thereby elevating venous return and cardiac output (Geevarghese et al.), and subsequently increasing blood pressure compensation in response to hemorrhage. Unfortunately, due to elevated pressure or hemodilution, these treatments concomitantly increase the risk of bleeding during non-compressible hemorrhage. Resuscitative endovascular balloon occlusion of the aorta (REBOA) is an endovascular hemorrhage control device that temporarily inflates a balloon in the aorta thereby restricting blood flow to the periphery while maximizing flow to vital organs such as the brain and heart. However, use of this device has been limited due to practical difficulties and deleterious effects such as ischemia, organ failure, and ischemia-reperfusion injury. Recently, a computational fluid dynamic model was created to simulate blood flow during different levels of aortic occlusion in hopes of better characterizing this technique and understanding its full implication on hemodynamics during hemorrhage (Renaldo et al.). Renaldo et al. calibrated a computational fluid dynamic model using pig data from anatomical (CT scans) and hemodynamic responses to hemorrhage. They found specific areas of high shear stress and low flow, potentially identifying areas at risk for thromboembolic complications. This model has translational value and could provide new insights when designing new endovascular devices or when training medical personnel on hemorrhagic control techniques.

Shock-induced damage to the vascular endothelium places critically ill patients at risk for abnormal coagulation profiles. This can range from a state of hypercoagulability and fibrinolytic shutdown that increases the risk for thrombosis, to a hypocoagulable state that is attributed to an overcompensation of anti-coagulant pathways to counterbalance the pro-thrombotic state. The review by Bunch et al. is the largest and most comprehensive review to date that describes these mechanisms across several forms of critical illness and shock, such as those attributed to hemorrhage, sepsis, burns, and hematologic malignancies. Their review further highlights that development of coagulopathy across these pathological states is commonly linked by a physiologic phenomenon known as Shock-Induced Endotheliopathy (SHINE). SHINE’s mechanism of action depends on increased sympathetic drive, which elevates circulating catecholamines and tissue plasminogen activator. As shock severity increases, this effect of SHINE becomes more profound to increase endothelial damage and subsequently the development of coagulopathy. Since patients can fluctuate between a hypo-and hypercoaguable states due to their etiology and severity of shock, the authors advocate for the use of viscoelastic tests such as thrombelastography and rotational thromboelastometry to monitor coagulation status and guide treatment.

To outline the pathophysiological process of shock, Dobson et al. presented a unified Systems Hypothesis of Trauma (SHOT) from central nerve system-cardiovascular coupling to endothelial-glycocalyx health, followed by mitochondrial integrity. The nerve-cardiovascular uncoupling, known as hemodynamic decompensation, is the initial step triggering SHOT and thus ought to be considered as the prior target for antishock treatment.

Trauma patient care continues to evolve, incorporating novel resuscitation methods and advanced techniques. Maintaining the balances among hemodynamics, hemostasis, and metabolism during compensatory regulations during shock is critical important. The current Research Topic of *Shock and Resuscitation* included novel methods and techniques that aim to to fill the current gap in understanding of the mechanisms that drive patient responses to trauma resuscitation, ultimately saving lives and improving quality of life after severe injuries.

## References

[B1] Clowes G. H. (1971). “Oxygen transport and utilization in fulminating sepsis and septic shock,” in Septic shock in man. Editor Hershey S. (Boston: Little, Brown and Co.), 85–106.

[B2] Crowell J. W. Smith E. E. (1964). Oxygen deficit and irreversible hemorrhagic shock. Am. J. Physiol. 206, 313–316. 10.1152/ajplegacy.1964.206.2.313 14120433

[B3] D'Alessandro A. Slaughter A. L. Peltz E. D. Moore E. E. Silliman C. C. Wither M. (2015). Trauma/hemorrhagic shock instigates aberrant metabolic flux through glycolytic pathways, as revealed by preliminary (13)C-glucose labeling metabolomics. J. Transl. Med. 13, 253. 10.1186/s12967-015-0612-z 26242576 PMC4523956

[B4] Dubin A. Kanoore Edul V. S. Caminos Eguillor J. F. Ferrara G. (2020). Monitoring microcirculation: Utility and barriers - a point-of-view review. Vasc. Health Risk Manag. 16, 577–589. 10.2147/VHRM.S242635 33408477 PMC7780856

[B5] Naumann D. N. Beaven A. Dretzke J. Hutchings S. Midwinter M. J. (2016). Searching for the optimal fluid to restore microcirculatory flow dynamics after haemorrhagic shock: A systematic review of preclinical studies. Shock 46 (6), 609–622. 10.1097/SHK.0000000000000687 27465755

[B6] Parrish D. Lindell S. L. Reichstetter H. Aboutanos M. Mangino M. J. (2016). Cell impermeant-based low-volume resuscitation in hemorrhagic shock: A biological basis for injury involving cell swelling. Ann. Surg. 263 (3), 565–572. 10.1097/SLA.0000000000001049 25915911 PMC4747844

[B7] Xiang L. Calderon A. S. Ryan K. L. Klemcke H. G. Mdaki K. S. Hudson I. L. (2023). Can polyethylene glycol-20k replace albumin for prehospital treatment of hemorrhagic shock when full resuscitation is unavailable? Shock 59 (5), 725–733. 10.1097/SHK.0000000000002099 36852970

